# Tumor hypoxia drives immune suppression and immunotherapy resistance

**DOI:** 10.1186/2051-1426-3-S2-P392

**Published:** 2015-11-04

**Authors:** Midan Ai, Pratha Budhani, Jie Sheng, Sadhana Balasubramanyam, Todd Bartkowiak, Ashvin R Jaiswal, Casey R Ager, Dhwani D Haria, Michael A Curran

**Affiliations:** 1 Department of Immunology, The University of Texas MD Anderson Cancer Center, Houston, TX, USA; 2 The University of Texas, Medical School at Houston, TX, USA; 3 Department of Nephrology, Second Affiliated Hospital, Dalian Medical University, Dalian, Liaoning, China

## 

Tumor hypoxia predicts poor outcomes across all cancers and has long been recognized as a critical source of resistance to both chemotherapy and radiotherapy. Despite the success of T cell immune checkpoint blockade in treating melanoma, aggressive adenocarcinomas of the prostate and pancreas are largely resistant to CTLA-4 and PD-1 antibody therapy in the mouse and in man. We find that hypoxic zones of these tumors resist infiltration by T cells even in the context of robust infiltration of T cells in normoxic areas of the same tumor (e.g. in the context of T cell checkpoint blockade). Beyond this lack of accessibility to tumor-specific T cells, hypoxia drives the establishment of a highly interdependent network of immunosuppressive stromal cells. Among these, we find myeloid-derived suppressor cells and myofibroblasts to be the critical populations which act together to suppress T cell responses and mediate immunotherapy resistance.

Evofosfamide is a hypoxia-specific chemotherapeutic pro-drug which is activated only in the hypoxic cores of tumors and thus can be co-administered with immunotherapy. We find that Evofosfamide-driven disruption of hypoxia zones sensitizes prostate cancer to antibody blockade of CTLA-4 and PD-1 in both transplantable and genetically-engineered murine models of prostate cancer. Co-administration of Evofosfamide and α-CTLA-4/α-PD-1 promotes tumor rejection in a significantly larger percentage of mice than either therapy alone. Mechanistic studies reveals that loss of immune resistance is a consequence of re-oxygenation of hypoxia zones which results in 1) loss of active myeloid suppressor cells, 2) reduced suppressive capacity of new myeloid immigrants, 3) loss of suppressive activation of myofibroblasts, and 4) enhanced infiltration of effector T cells. Therefore, this combination of hypoxia disruption and T cell checkpoint blockade has immense potential to render some of the most therapeutically resistant cancers sensitive to immunotherapy.

